# Immunostaining in whole-mount lipid-cleared peripheral nerves and dorsal root ganglia after neuropathy in mice

**DOI:** 10.1038/s41598-019-44897-7

**Published:** 2019-06-10

**Authors:** L. Bernal, E. Cisneros, N. García-Magro, C. Roza

**Affiliations:** 1 Department of System’s Biology, Medical School, University of Alcala, Alcalá de Henares, 28871 Madrid, Spain; 2 Centro Universitario Internacional de Madrid (CUNIMAD), Madrid, Spain; 3 Department of Anatomy, Histology and Neuroscience, Medical School, Autonoma University of Madrid, 28029 Madrid, Spain

**Keywords:** Somatic system, Neurophysiology

## Abstract

Immunohistochemical characterization of primary afferent fibers (intact or after nerve damage) is traditionally performed in thin sections from dorsal root ganglia (DRGs) or in teased fibers, as light scattering in whole-mounts compromises visualization. These procedures are time-consuming, require specific equipment and advanced experimental skills. Lipid-clearing techniques are increasing in popularity, but they have never been used for the peripheral nervous system. We established a modified, inexpensive clearing method based on lipid-removal protocols to make transparent peripheral nerve tissue (inCLARITY). We compared retrograde-labeling and free-floating immunostaining with cryo-sections. Confocal microscopy on whole-mount transparent DRGs showed neurons marked with retrograde tracers applied to experimental neuromas (Retrobeads, Fluoro-ruby, Fluoro-emerald, DiI, and Fluoro-gold). After immunostaining with calcitonin gene-related peptide (peptidergic) or isolectin IB4 (non-peptidergic), nociceptors were visualized. Immunostaining in transparent whole-mount nerves allows simultaneous evaluation of the axotomized branches containing the neuroma and neighboring intact branches as they can be mounted preserving their anatomical disposition and fiber integrity. The goal of our study was to optimize CLARITY for its application in peripheral nerve tissues. The protocol is compatible with the use of retrograde tracers and improves immunostaining outcomes when compared to classical cryo-sectioning, as lack of lipids maximizes antibody penetration within the tissue.

## Introduction

Peripheral afferents are pseudounipolar neurons which cell bodies (located in the dorsal root ganglia -DRG- or trigeminal ganglia -TG-) give rise to a central axon that goes into the central nervous system and a peripheral axon that ends in the skin, muscles or visceral organs among others. As they convey sensory information, any lesion or disease affecting these neurons lead to the perception of abnormal sensations (typically spontaneous and/or evoked pains) termed neuropathic pain. Each of these symptoms depends on specific pathophysiological mechanisms. For example, spontaneous burning pain is likely dependent on spontaneous activity from hyper-excitable nociceptors and regenerating sprouts^1–3^ from either damage or neighboring intact afferents, although their specific roles are poorly understood^4–7^. Electric-shock like-sensations (paroxysmal pain) seem to arise from high-frequency bursts in demyelinated non-nociceptive Aβ-fibers^1^. Recent advances in the treatment of neuropathic pain are based on patient’s “sensory-abnormalities” rather than the etiology of the neuropathy^8,9^. Such a mechanism-based approach requires the identification and characterization of each of the peripheral fibers involved.

Retrograde tracers such as those commonly used in neurobiology to map neuronal networks can also identify damaged and intact primary afferents after nerve trauma. For example, carbocyanines (DiI), dextran conjugates (Fluoro-ruby, FR, and Fluoro-emerald, FE) or Fluoro-gold (FG) are frequently used when studying peripheral nerve regeneration^10–14^. Others, like fluorophore-conjugated latex microspheres (retrobeads, RB), have been less explored despite their limited spread, fast diffusion time and minimum fading^15–17^. On the other hand, immunohistochemistry (IHC) allows to classify functional populations of unmyelinated pain C-fibers in DRGs or TGs based on the presence of calcitonin-gene related peptide (CGRP) or substance P (SP) (peptidergic neurons) and staining with isolectin IB4 from Griffonia simplicifolia (non-peptidergic neurons)^18^. At the peripheral level, the expression of myelin proteins and different sodium and potassium channels changes after nerve damage^19–21^. The detection of fluorescence markers is usually performed by epi- or confocal microscopy on thin cryo-sectioned slices or teased nerve fibers obtained from fixed tissues^20–23^ which exclude the possibility to evaluate the three-dimensional organization.

In recent years, there has been a growing interest in optimizing methods for molecular characterization in transparent tissues. One of the first descriptions dates from 2003 when brains of invertebrate animals were made transparent by using the expensive mounting medium FocusClear®^24^. Up to now, the most popular clearing technique applied to vertebrate’s central nervous system is CLARITY^25^, and similar methods have been developed and applied to other tissues such as organs, bone or muscle^26–29^. Improvements in the original protocol included passive clearing methods (i.e. PACT, mPACT)^27,30^, improved optical clearing^31^ or acrylamide-free clearing (i.e. 3DISCO, BABB, ethyl cinnamate, Scale)^32–36^. All these techniques remove lipids while keeping structural proteins intact, which ensures higher light penetration under the microscope and allows for three-dimensional (3D) reconstructions. Combination of clearing techniques with IHC protocols has been achieved with different antibodies^25^. It is also possible to study specific axonal tracts and their development, as shown in mouse embryos or adult mice^33,37^. Detection of lipophilic carbocyanines (i.e. DiI analogs and FM 1-43FX) and viral vectors is compatible with PACT^38,39^. Cholera-toxin subunit B seems to be compatible with 3DISCO^40^ and protocols based on organic solvents works well with dextrans^33^. However, the introduction of these approaches in research laboratories is still scarce due to their high cost, the complexity of published protocols or possible negative impact on the fluorescence signal.

As no one has yet aimed to apply these protocols specifically to the peripheral nervous system, here we have adapted an affordable and simplified passive lipid-clearing protocol, which can be easily introduced in any laboratory. We have shown its suitability for the study of the alterations observed in the peripheral nervous system upon damage, but we believe it might be adapted for other experimental contexts.

## Results

### Efficient and inexpensive passive clearing of peripheral nervous tissues

**Figure 1:**
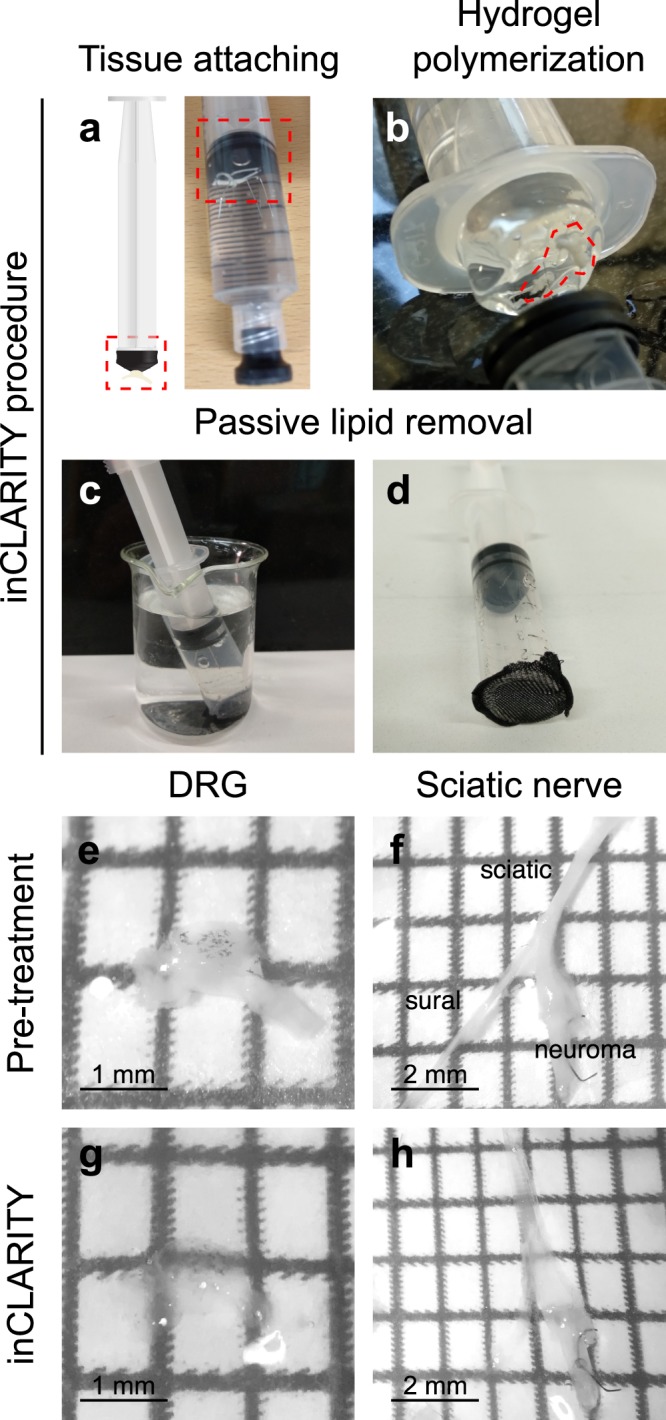
inCLARITY protocol for imaging intact peripheral nervous tissue. (**a**) Original photographs of the experimental arrangement showing how tissue samples get attached to the syringe plunger with minutien pins (dotted square). The corresponding syringe was then filled with the hydrogel solution and closed to keep an oxygen-free environment. (**b**) After polymerization, the samples embedded in the hydrogel (red circle) were carefully removed and re-attached to the plunger. (**c**) The plungers were inserted into modified syringes for passive lipid removal. (**d**) Note the nylon mesh at the open end of the syringe that avoids mixing of samples when processed together. (**e**–**h**) Representative low-magnification photographs of a DRG and a sciatic nerve before (**e**,**f**) and after clearing (**g**,**h**). The black dots in the DRG (**e**,**g**) correspond to epithelial pigments characteristic of C57/Bl6 mice. No obvious changes in volume or structure were noticeable.

The protocol presented here is suitable to make transparent tissue samples of mice peripheral nervous system allowing for efficient immunostaining. Alternative preparation of the samples requires time-consuming procedures such as cryo-sectioning (DRG and TG) or teasing nerve fibers. Protocol steps are summarized in Fig. 1a–d. The time required to get satisfactory transparent tissues is proportional to the sample size, hence, ~1 mm DRGs needs 7 days and increases to 20 days for ~6 mm TG. Nevertheless, the total working time is reduced to <1 hour. As shown in Fig. 1e–h and Supplementary Fig. 1a,b no obvious changes in volume or structure occur after clearing. The polymerization process can be reduced by up to 2.5 hours by increasing incubation temperature (up to 40 °C) and lipid-clearing to half incubation-time when performed at 37 °C. Based on the quantity of specific reagents used, the estimated price of the inCLARITY protocol was ~10 € per mouse (4–6 DRGs and 2 sciatic nerves).

### Retrograde tracers in whole-mounts of transparent DRGs

**Figure 2:**
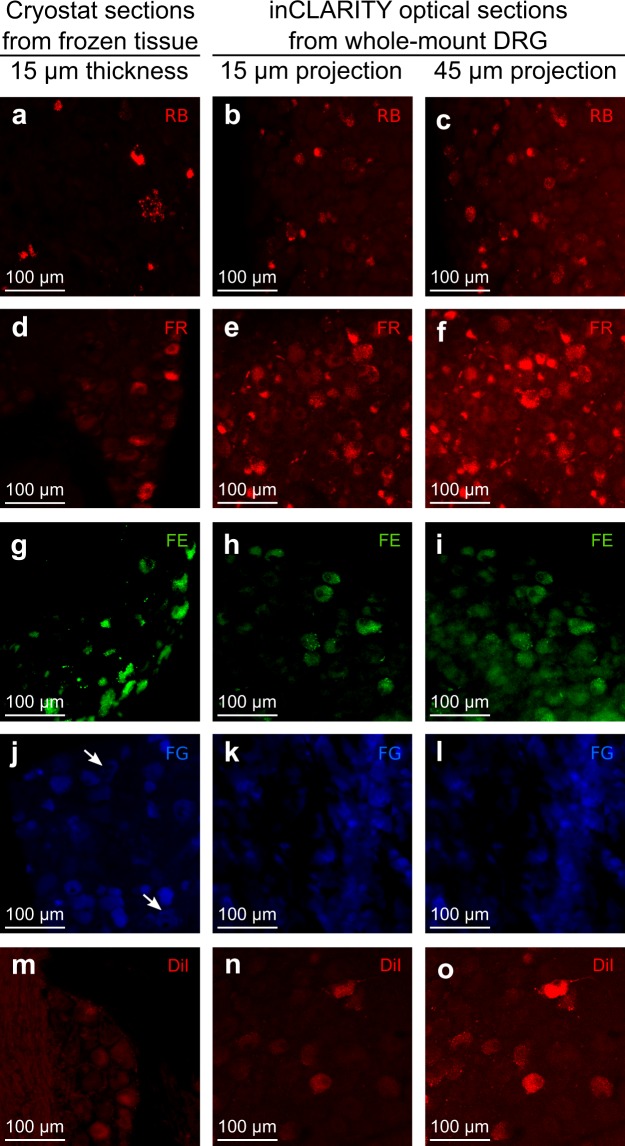
Retrogradely labeled neurons are visible in DRGs after inCLARITY. Epifluorescence images of 15 μm cryostat sections -left column- and confocal optical sections (15 or 45 μm sum intensity projection) of transparent whole-mount DRGs –middle and right columns-. Neurons were marked with different retrograde tracers that were applied during SNI surgery at the neuroma: RB (**a**–**c**), FR (**d**–**f**), FE (**g**–**i**), FG (**j**–**l**) and DiI (**m**–**o**). Note that some neurons marked with FG present vacuolated cytoplasm (arrows in j).

**Figure 3:**
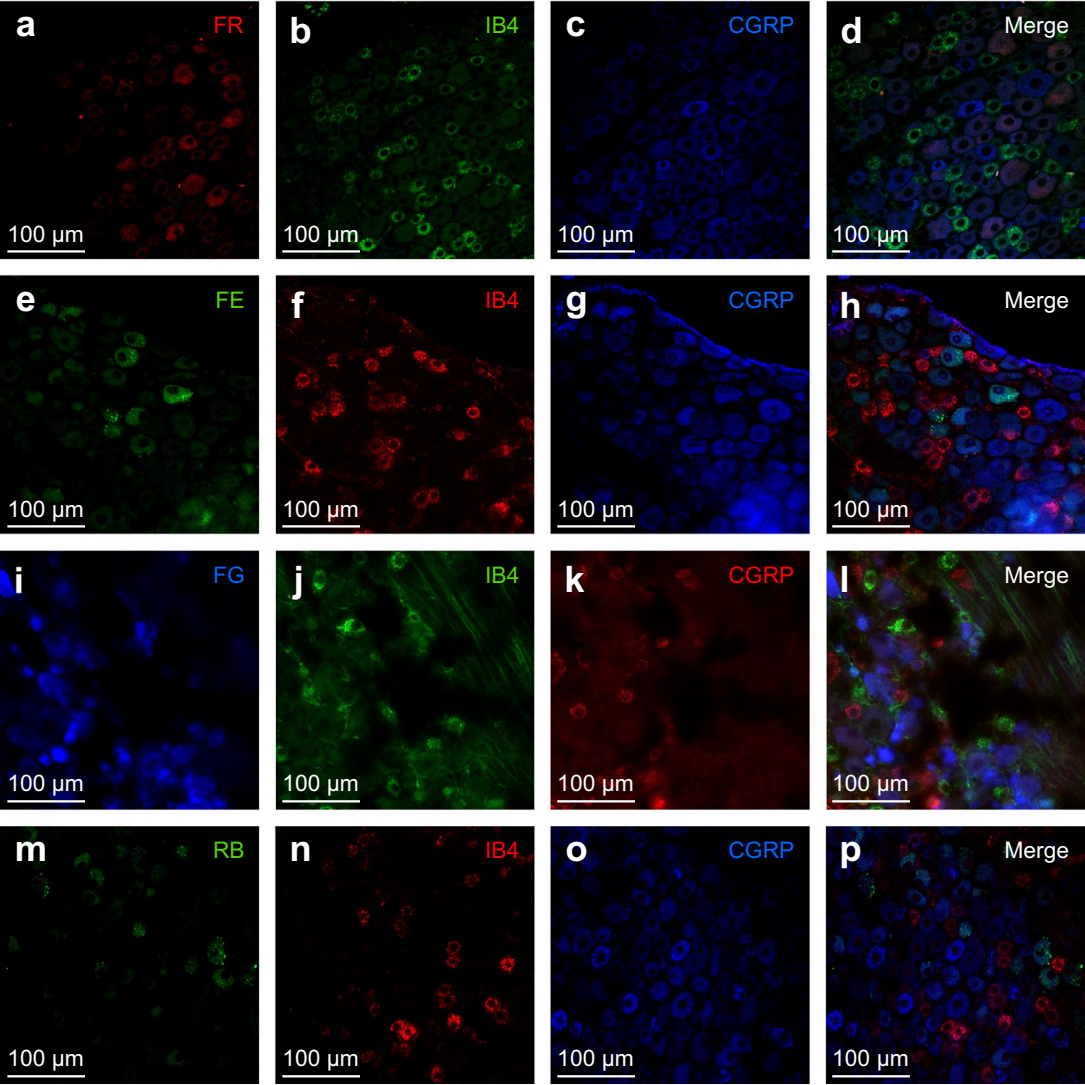
Immunohistochemistry after inCLARITY in whole-mount DRGs. Double immunofluorescence with IB4 (second column; **b,f,j,n**) and CGRP (third column; **c,g,k,o**) in transparent whole-mount DRGs with retrogradely labeled neurons (first column). FR, FE or FG were applied at the neuroma (**a,e,i**, respectively), hence, these stained cell bodies correspond to axotomized neurons. gRB were applied subcutaneously at the hind-paw (**m**), hence, the stained cells represent intact neurons.

All the retrograde tracers applied to the neuroma were observed at the DRGs after 1 or 2 weeks both in cryo-sections and after inCLARITY (see Figs 2 and 3). Among those, red RB (rRB) showed the best signal-to-noise ratio for both procedures (Fig. 2a–c). In DRG cryo-sections, FR was only visualized after immunostaining against tetramethylrhodamine (Fig. 2d) and FE signal was very low (Fig. 2g). However, both FR and FE showed stronger signals in DRG cells after inCLARITY without further processing (Fig. 2e–f,h–i). For both procedures, FG conspicuously marked several cells and occasionally some showed apparent vacuolated cytoplasm, which might indicate cellular damage (Fig. 2j). On the contrary, very few cells were marked with DiI, but its signal was better in transparent DRGs (Fig. 2m–o). Green RB (gRB) applied subcutaneously in the dorsal hind paws, were visualized after 2 weeks but only in transparent DRGs (Fig. 3m). These comparisons are summarized in Table 1.

**Table 1:** Characteristics of the retrograde tracers and detection on cryo-sections and inCLARITY processed samples.

Dye	Transported by	Application	Excitation (nm/color)	Emision (nm/color)	Source	DETECTION	
						Cryo-sections	inCLARITY
*Fluoro-ruby (FR)*/*Dextran Tetramethyl-rhodamine*	Damaged fibers	Crystal on top of the neuroma	540–552/green	590/orange	Invitrogen (D1817)	Require IHC for tracer visualization	Direct visualization
*Fluoro-emerald (FE)*/*Dextran Fluorescein*	Damaged fibers	Crystal on top of the neuroma	450–490/blue	515/green	Invitrogen (D1820)	Low signal (exposure times > 700 ms)	Strong signal (exposure times ~200–300 ms)
*Retro-Beads IX (RB)*	Damaged/ undamaged fibers?	Intraperineural injection (red) Intraepidermal injection (green)	530/green 460/blue	590/orange 505/green	Lumafluor	Low signal (detection from 20x magnification)	Strong signal (detection from 10x magnification)
*Fluoro-Gold (FG)*/*2-hydroxystilbene 4*,*4*′*dicarboxamidine bis methasulfonate*	Damaged/ undamaged fibers	Intraperineural Injection	365/UV	420/violet	Thermo-Fisher (H22845)	Good signal-to-noise ratio	Good signal-to-noise ratio
*DiI*/*1*,*1*′*-Dioctade-cyl-3*,*3*,*3*′,*3*′*-Tetra-methylindocarbo-cyanine Perchlorate*	Damaged/ undamaged fibers	Crystal on top of the neuroma	540–552/green	590/orange	Sigma-Aldrich (42364)	Good signal-to-noise ratio	Good signal-to-noise ratio

### Immunostaining in whole-mounts of transparent DRGs and nerves

In order to evaluate the compatibility of inCLARITY with the staining of sensory neurons, we tested IB4 labeling and CGRP immunostaining on DRGs. The concentration of the markers previously used on frozen cryo-sections was suitable for transparent samples in whole-mounts, although incubation time was increased for better results.

Figure 3 shows multiple fluorescence signals in transparent DRGs. CGRP and IB4 are distinctive markers for peptidergic and non-peptidergic C-nociceptors, respectively^41^; as expected, no double staining for these markers was observed. Trigeminal ganglia, which are significantly larger than DRG, were also satisfactorily stained using this procedure (Supplementary Fig. 1c). We show here that inCLARITY permeabilized both DRGs and TGs, as staining was visible in optical sections from the innermost tissue, allowing for three-dimensional reconstruction (see Fig. 3, Supplementary Fig. 1 and Supplementary Fig. 2). Furthermore, cryo-sectioning of immunostained transparent DRGs confirmed homogenous antibody penetration within the DRG (n = 7 DRGs, Supplementary Fig. 3). In clear contrast, staining for IB4 in non-transparent whole-mount DRGs was unsuccessful (n = 5, Supplementary Fig. 3).

**Figure 4:**
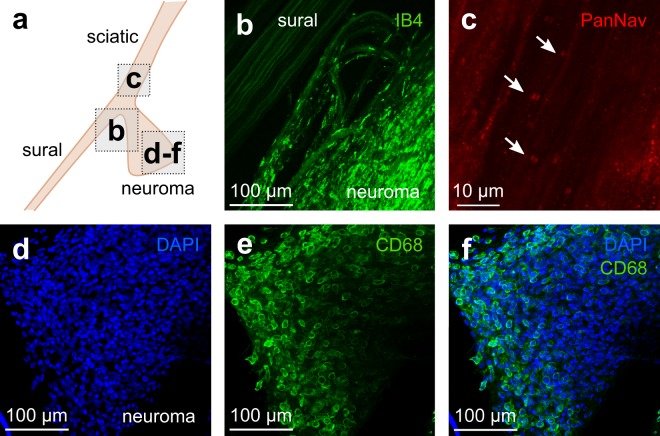
Immunohistochemistry after inCLARITY in whole-mounts of peripheral nerves. (**a**) Scheme of a nerve-end neuroma (tibial and peroneal branches) together with the intact sural branch. (**b**) Single optical section of a whole-mount nerve/nerve-end neuroma labeled with IB4 showed clear structural differences between intact fibers running within the sural nerve and damaged fibers within the neuroma. (**c**) Single optical section from the common nerve area in which PanNav staining revealed the presence of nodes of Ranvier (arrows). (**d**–**f**) In the neuroma, many of the cell nuclei (DAPI, blue) belonged to macrophages (CD68, green) as shown in this 95 μm maximum projection.

The inCLARITY protocol was also useful to get an overview of the events that take place during axonal damage and repair of peripheral nerves. Following spared nerve injury (SNI), whole preparations containing the neuroma together with the intact sural nerve were processed for IHC (see scheme in Fig. 4a) in order to get a simultaneous examination of damaged and intact branches. As revealed with IB4 staining, the chaotic structure of the fibers in the neuroma contrast with the preserved structure of the intact branch (Fig. 4b). Furthermore, the inCLARITY protocol is compatible with the detection of nerve compartments, such as nodes (Fig. 4c), and the immune cells that invade the injured tissue (Fig. 4d–f).

## Discussion

In the present study, we have successfully adapted an affordable method to make transparent peripheral nervous system tissues. Removal of lipids allows for the detection of fluorescent signals in whole-mounts. In the context of neuropathic pain, retrograde tracers help to identify the cell bodies of damage vs intact axons, and specific antibodies target different subsets of nociceptive C-fibers. Our inCLARITY protocol improves classical immunostaining in cryo-sections and teased fibers. We believe that this method can be implemented in any laboratory without special requirements or great expenses.

The different retrograde tracers used here –dextrans, microspheres, DiI and FG- preserved their fluorescent signal after inCLARITY, indicating that, despite their chemical nature, all are fixable and remain in the tissue after the passive protocol used here. Both in cryo-sections and after inCLARITY, red and green RB were visualized. RB produce a distinct granular pattern in the neuronal cytoplasm highly resistant to fading and no diffusion to neighboring cells. Although RB preferably label disrupted axons^16^, in our present study we observe stained cell bodies at the DRGs when RB were applied at the peripheral terminals in the intact skin. We could speculate that some axons could have been damaged during the injection procedure favoring dye uptake. On the other hand, RB might penetrate peripheral terminals. Regardless of the mechanisms, we show here that RB taken up by peripheral terminals travel retrogradely to their cell bodies which display a strong fluorescence intensity.

The fluorescence dextran-conjugates FR and FE are only taken up by disrupted axons^42^. In comparison with the strong signal produced in whole-mounts processed by inCLARITY, under our experimental conditions, dextran conjugates were barely detected in cryo-sections, and FR was only detected after immunostaining against tetramethylrhodamine. Thus, inCLARITY improves fluorescence visualization and avoids additional immunostaining steps^43,44^. As fixation time was the same for both procedures, one might speculate that preservation of fluorescence is compromised during cryo-sectioning. FG (a water-soluble crystalline tracer) is commonly used for retrograde/anterograde labeling due to the massive axonal uptake and bright fluorescence of cell bodies. However, we observed in both cryo-sections and whole-mounts, several cells with vacuolated cytoplasms, which, in accordance with previous reports, points to neurotoxic effects^45,46^. Compatibility of DiI (lipophilic carbocyanine) analogs with clearing methods was previously reported by using PACT^38^. In our experimental conditions, DiI signal was slightly better after inCLARITY. However, previous reports using DiI have shown cytotoxic effects and large diffusion to unlabeled cells *in vivo*^47^ or after sectioning^48^.

Researchers should evaluate the suitability of each of these dyes according to their experimental purposes, but in view of our results, the use of dextrans or retrobeads are recommended to achieve the strongest fluorescence signal without apparent cytotoxic effects and accurate distinction of the origin of the fiber.

Our data also show that after inCLARITY, immunostaining is feasible in whole-mount DRGs and peripheral nerves. Other inexpensive clearing methods have been described (i.e. ethyl cinnamate, BABB)^34,35^, but these are based on organic solvents and require dehydration which would only allow immunostaining by intravenous injection of antibodies before fixation of the animal. Moreover, one of the limitations of immunostaining can be the price of the antibodies. In our protocol, the amount of antibody required to stain a whole-mount DRG is the same as that used for one slide containing DRGs cryo-sections or teased fibers (~200 µl). Although it was not tested here, it could be possible to perform several rounds of immunostaining with different antibodies^49^. In transparent DRGs the evaluation of fluorescent signals can be performed in whole mounts, hence cell counting is possible on optical sections of adjustable thickness. Optical sectioning provides a simple, fast and reasonable alternative when aimed to obtain cellular resolution and 3D reconstructions in which automatic counting could be implemented when sharp staining. Although the working distance of the objectives limits the deepness of the imaging, compromising full 3D reconstructions, it is now possible to use long working distance objectives, and/or light-sheet or two-photon microscopes.

In comparison, traditional processing of tissue samples for immunostaining requires serial sectioning and microscopic analysis of that sections all of which are time-consuming. In addition, handling peripheral nervous tissues usually requires working with thin sections which are delicate and difficult to obtain, requiring experimental practice and the use of specific devices (i.e. cryostat or microtome) which might not be present in all laboratories interested in the study of the peripheral nervous system. Occasionally, frozen tissues affected by the formation of ice crystals, could disturb sample size or structure, sub-cellular details and negatively impact immunostaining.

Transparent thin peripheral nerves and neuromas can be examined as whole-mounts in which the general anatomy and structure are preserved. This improves the study of cellular and molecular changes that occur after nerve damage along the structure of the nerve. Unfortunately, only a few studies have explored changes that occur at the damaged area in experimental models of neuropathic pain, mostly because proper visualization after immunostaining can be only achieved when performed in teased fibers^19,22,23,50^, which is extremely tedious, time-consuming and produces a loss of the general vision. In addition, neuromas are impossible to tease due to the amount of debris, and accumulation of scar tissue/cells among others. inCLARITY avoids both sectioning and teasing, but, more important, allows to keep the 3D structure. Thus, fibers belonging to neuroma or intact nerve can be accurately identified and examined.

In summary, we have successfully adapted a lipid-clearing method to make transparent peripheral nervous tissue that offers several advantages in comparison with traditional cryo-processing. We have proved its compatibility with different retrograde tracers commonly used in neurobiology and with immunostaining. We strongly recommend the use of this methodology for the study of peripheral neurons with immunohistochemical approaches.

## Material and Methods

Adult outbred C57BL6 mice of both sexes (n = 29, mean body weight 22 ± 0.8 g) bred at the University Animal House were used. European Union and State legislation for the regulation of animal experiments was followed. All experimental protocols were approved by the University of Alcala Committee on Animal Research and the Regional Government (project license: ES280050001165).

### Induction of neuropathy and retrograde labeling

Spared nerve injury -SNI- was performed in 22 animals as previously described^51^. During the surgical procedures, the mice underwent deep anesthesia with ~2–3% isoflurane in 100% oxygen. Briefly, an incision was made in the skin on the lateral surface of the thigh and the sciatic terminal branches (sural, common peroneal and tibial nerves) were exposed. The common peroneal and the tibial nerves were ligated with 8/0 silk and sectioned distal to the ligature. The sectioned end was inserted into an approximately 2 mm long silicone tube (0.45 mm internal diameter) to prevent lateral innervation of surrounding tissue. The tube was tied in place with the ligature silk and the incision was closed in layers (5/0 sutures for the muscle and surgical staples for the skin). FG (4% in distilled water) or rRB (~1 μl) were collected by capillarity with a glass micro-pipette (~20 μm diameter tip), which was then used to inject the dye intra-perineurium using positive pressure through a cannula connected to the micro-pipette. FR, FE, and DiI (1,1′-dioctadecyl-3,3,3′3′-tetramethylindocarbo-cyanine perchlorate) crystals were placed directly inside the tube containing the neuroma, whose end was closed with surgical grease (Fine Science Tools). Retrograde labeling was performed in three to five animals per tracer. In two of the animals, gRB were injected subcutaneously in the hairy skin of the contralateral paws with a glass-pipette (through a hole previously opened with a 30G needle).

Animals were then housed and inspected periodically for infections or abnormal behavior. The mice had access to water and food *ad libitum*.

The characteristics of the retrograde tracers are shown in Table 1.

### Tissue collection

Tissues were collected 1 or 2 weeks after surgery. The mice were deeply anesthetized with a mixture of ketamine (75 mg/kg; Imalgene 500, Merial Laboratorios) and xylazine (12 mg/kg; Rompun, Bayer) and perfused transcardially with 4% (w/v) paraformaldehyde (PFA) in phosphate buffer (PB) 0.1 M, pH 7.2. Nerve end neuromas, L3 and L4 DRGs from both ipsilateral and contralateral sides were extracted and post-fixed in the same solution overnight at 4 °C. Additionally, trigeminal ganglia were extracted from three of the animals and processed likewise.

### Preparation and immunostaining of frozen tissue sections

To assess the viability of retrograde tracers, we processed some DRGs by cryo-sectioning. Samples were washed with PB 0.1 M three times and cryo-protected with 30% sucrose in PB (overnight, 4 °C). The tissue was embedded in OCT (Tissue-Tek®) and deep-frozen until use. DRGs were cryo-sectioned at 15 µm (Leica CM1950; Leica, Wetzlar, Germany) and mounted in Mowiol®. Slices were visualized and photographed under fluorescence microscopy (Olympus BX61, Tokyo, Japan).

To allow FR visualization immunostaining was performed as follows: the sections were incubated in blocking solution (10% donkey serum in Tris-buffered saline, TBS) for 2 h and permeabilized in 0.5% Triton X-100 in TBS for 10 min. Then, they were incubated with anti-tetramethylrhodamine antibody (Molecular Probes, 1:200) diluted in TBS at room temperature (RT) for 16 h. Sections were washed in 0.2% Tween 20 in TBS and incubated with Alexa-Fluor 594-conjugated donkey anti-rabbit secondary antibody (Jackson ImmunoResearch Laboratories, Inc., West Grove, PA, USA; 1:500) at RT for 1.5 h. Sections were then mounted in Mowiol®.

### Passive lipid-clearing protocol

The tissue samples were fixed to a Sylgard® base attached to the final edge of a 10 ml syringe plunger and then introduced in the syringe, which was filled with ~5 ml of the hydrogel solution (4% acrylamide, 0.13% bis-acrylamide, 0.25% (w/v) V-50 in 4% PFA in PBS pH 7.4) and kept for 2 hours at 4 °C. The mixture acrylamide/bis-acrylamide 29:1 (40%) was purchased from National Diagnostics and V-50 (2,2′-Azobis(2-methylpropionamidine) dihydrochloride) from Sigma Aldrich. Air bubbles were carefully removed, and the syringe was closed with a plug to keep the solution oxygen-free. Then, the syringe was transferred to a 37 °C bath with agitation for 4 h to allow polymerization. The plunger was then detached, and the gel carefully removed to free the tissue samples which were then incubated in the clearing solution (8% sodium dodecyl sulfate -SDS- in PBS 0.1M, pH 7.4) at RT for 7–12 days to ensure lipids removal. Two daily SDS-exchanges accelerated the process. SDS was purchased from Bio-Rad. The tissue samples, which remained attached to the plunger edge during the whole procedure, were finally washed for 1 day in PBS 0.1M and mounted with Mowiol® (refractive index 1.41–1.49, Sigma-Aldrich). As the protocol is inexpensive, we call it inCLARITY. The protocol is depicted in Fig. 1a–d and summarized in Table 2. Photographs before and after inCLARITY were taken with an iPhone 7 through the ocular of a Nikon SMZ-2B scope.

**Table 2:** Summarized inCLARITY protocol.

	Fixation	Hydrogel polymerization	Lipid-clearing	Wash	IHC	Wash	Mounting
*Procedure*	Transcardial PFA 4%-fixation	Acrylamide/Bisacrilamide + V-50 in 4% PFA in PBS, pH 7.4	8% SDS, pH 7.4	PBST 0.1%, pH 7.4	Primary & secondary antibodies in PBST 0.1%	PBST 0.1%, pH 7.4	Mowiol
*Time*	<1 h Post-fixation PFA 4% Overnight 4 °C	2 h at 4 °C followed by 4 h at 37 °C	7–20 days at RT	1 day at RT	5–8 days at RT	1 day at RT	Until dry

### Immunostaining after lipid-clearing

After removal of lipids, tissue samples were washed free-floating in PBST (0.1% Triton X-100 and 0.01% sodium azide in PBS 0.1M) for 1 day and incubated with specific markers prepared in PBST for 5 days at RT. Putative nociceptive neurons from DRGs were identified by using IB4 and CGRP antibody. Nerve fibers were marked with IB4; cells localized in the neuroma were identified with the antibody against cluster of differentiation 68 (CD68, macrophage lineage) and DAPI (nucleus). Samples were washed in PBST for 1 day at RT and then incubated with the following secondary antibodies (1:500, in PBST 0.1%, 2 days): Alexa-488 conjugated donkey anti-rabbit/mouse/goat, Alexa-594 conjugated donkey anti-rabbit/mouse/goat or Alexa-405 conjugated donkey anti-goat (as appropriate). Secondary antibodies were purchased from Jackson ImmunoResearch (UK). Samples were washed in PBST for 1 day at RT and mounted in Mowiol®. Fixed L3/L4 DRGs were processed in parallel (free-floating IHC in whole-mount) to test antibody penetration without clearing.

The optimal concentration of antibodies and incubation time for immunostaining after inCLARITY were set upon testing IB4-FITC in DRGs (1:250 for 3 and 5 days or 1:500 for 5 days). The characteristic of the primary antibodies used, the manufacturer’s information and concentration are summarized in Table 3.

**Table 3:** Characteristics of the primary antibodies.

Antibody	Immunogen	Raised in, mono- vs. polyclonal	Manufacturer	Dilution	Incubation time	
					cryosections	inCLARITY
*IB4-FITC*		*Griffonia simplicifolia*	Vector (FL-1201)	1:500	16 h	5 days
*IB4-Alexa594*		*Griffonia simplicifolia*	Vector (DL-1207)	1:500	16 h	5 days
*CGRP*	C-terminus of CGRP protein	Goat. Polyclonal	Santa Cruz Biotechnology (sc-8857)	1:200	16 h	5 days + 2 days secondary Ab
*Pan Nav*	1501–1518 aa residues of vertebrate Nav1.1	Rabbit. Polyclonal	Alomone Labs (ASC-003)	1:500	16 h	5 days + 2 days secondary Ab
*CD68*	Single chain glycoprotein of 110 kDa expressed on the lysosomal membrane of myeloid cells	Mouse. Monoclonal	Abcam (ab-31630–125)	1:400	16 h	5 days + 2 days secondary Ab
*Tetramethyl-rhodamine*	Tetramethylrhodamine fluorophore (FR)	Rabbit. Polyclonal	Molecular probes (A6397)	1:500	16 h	—
*DAPI*	DNA	—	Sigma-Aldrich (D9542)	1:1000	5 min	16 h

### Microscopy and image analysis

Whole-mount samples were observed in a Leica TCS SP5 Confocal Microscope and images were taken using a dry 10X objective or oil immersion 20X and 40X objectives. The images were acquired at 1024 × 1024 pixels and image stacks were obtained with the Leica LAS AF software. We used those acquisition parameters that produced better signal-to-noise ratio as sample comparisons in terms of fluorescence intensity was out of scope in this study.

All images were analyzed and processed using Fiji software^52^ and GIMP 2.8.22 software. For cryo-sections, fluorescence microscope images correspond to 15 µm sections. In DRG experiments, confocal images correspond to single optical sections or 15 µm and 45 µm sum intensity projections. In nerve experiments, confocal images correspond to 95 µm maximal intensity projections. The 3D visualization shows a partial reconstruction up to 150 µm of a DRG.

## Supplementary information

Supplementary Figures
