# Supplementary material for: Immunostaining in whole-mount lipid-cleared peripheral nerves and dorsal root ganglia after neuropathy in mice
Source: Sci Rep. 2019 Jun 10;9:8374. doi: 10.1038/s41598-019-44897-7 (PMC6558043; doi:10.1038/s41598-019-44897-7)
Supplement: Supplementary file 1 — Supplementary Figures [file 41598_2019_44897_MOESM1_ESM.pdf]

Immunostaining in whole-mount lipid-cleared peripheral nerves and dorsal root ganglia after neuropathy in mice

L. Bernal[1] , E. Cisneros[1,2], N. García-Magro[3] , and C. Roza[1] *

[1] Department of System's Biology. Medical School, University of Alcala. Alcalá de Henares, 28871 Madrid, Spain.

[2] Centro Universitario Internacional de Madrid (CUNIMAD), Spain.

[3] Department of Anatomy, Histology and Neuroscience, Medical School, Autonoma University of Madrid, 28029 Madrid, Spain.

Corresponding author: Carolina Roza

Dpto. Biología de Sistemas

Edificio de Medicina - Campus Universitario

Universidad de Alcalá, Madrid, 28871 Spain

Telephone + 34 91 885 4595

E-mail carolina.roza@uah.es

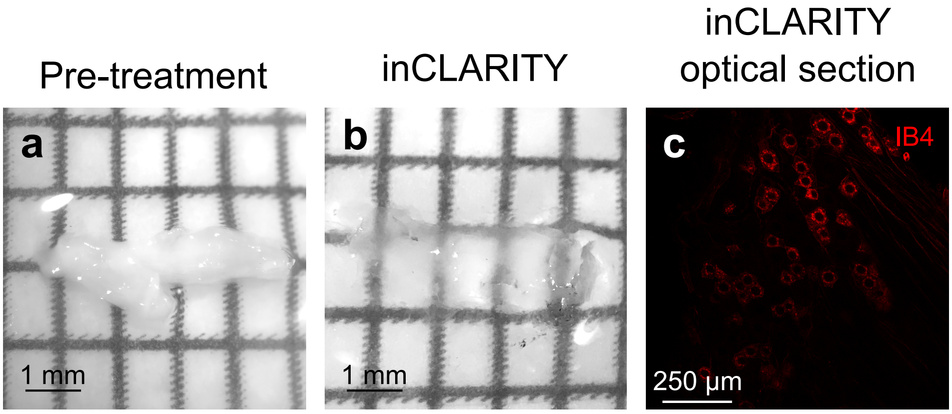

Supplementary Figure 1. Immunostaining after inCLARITY in Trigeminal Ganglia. Example of a TG before (a) and after (b) 20 days of clearing. (c) Single optical section from a whole-mount TG that was stained with IB4. Note that large tissue samples were also suitable for whole-mount imaging after this procedure.

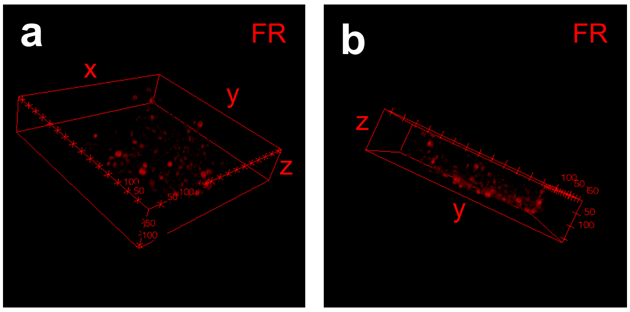

Supplementary Figure 2. Partial 3D reconstruction of a retrogradely labeled DRG after inCLARITY. Z-stack of a whole-mount DRG with neurons retrogradely labeled with FR applied at the neuroma (hence, marking the cell bodies of axotomized fibers). The DRG was imaged using a dry 10X objective. Values on x- and z-axis indicate size in micrometers.

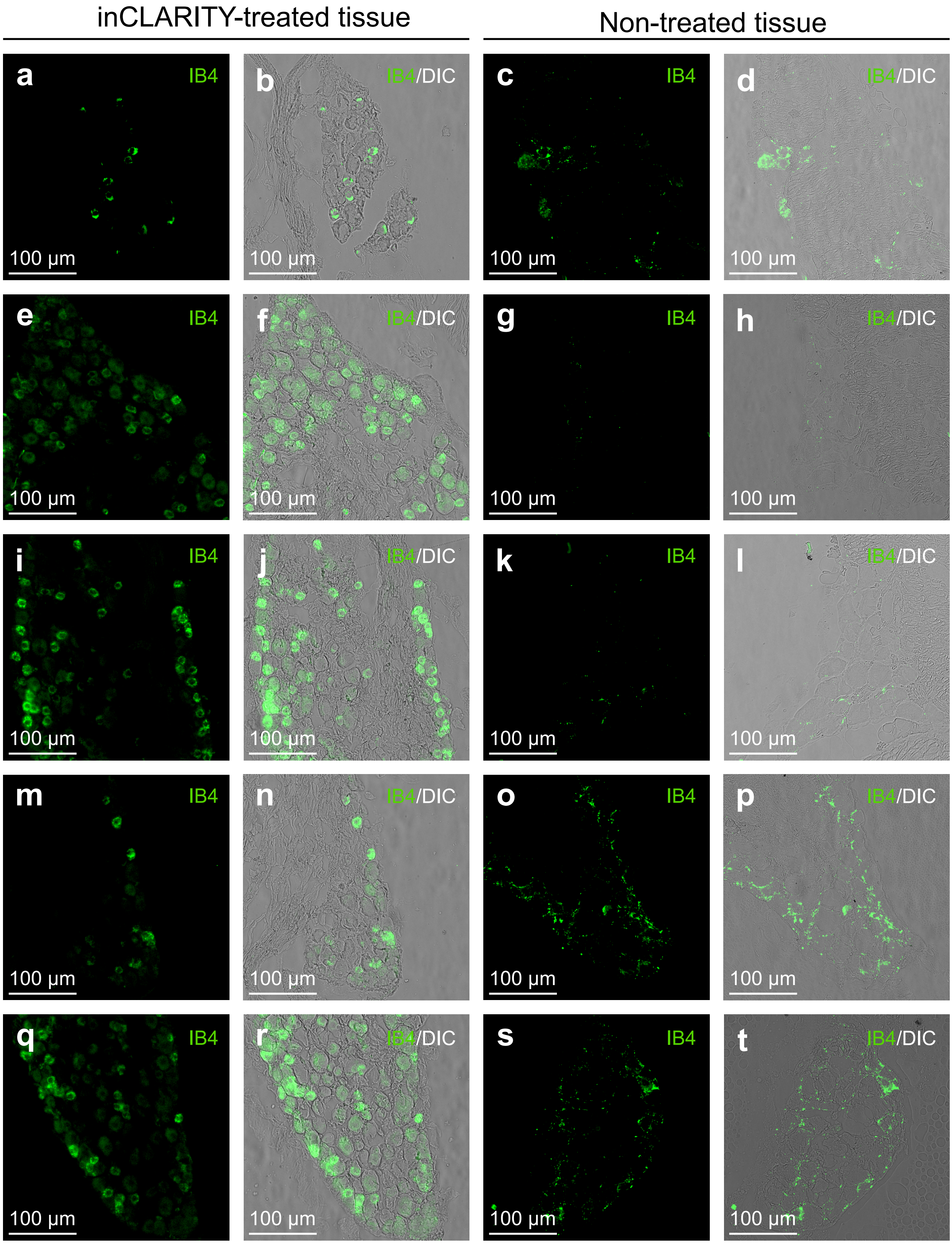

Supplementary Figure 3. Immunostaining of whole DRGs is only feasible after inCLARITY. Epifluorescence images of cryostat serial sections (15 μm) obtained from a wholemount DRG stained for IB4 after inCLARITY (left columns) and a whole-mount DRG staining for IB4 without the clearing process (right columns). After inCLARITY, IB4+ neurons were visualized along the whole DRG, indicating full marker penetration. On the other hand, in non-transparent DRGs, IB4 was unable to penetrate hence, no positive cells were staining, despite transmitted light (DIC) images revealed the presence of cells bodies.
